# Variety-Seeking Behavior in Consumption: A Literature Review and Future Research Directions

**DOI:** 10.3389/fpsyg.2022.874444

**Published:** 2022-06-06

**Authors:** Yuan Zhang

**Affiliations:** Business School, Huaqiao University, Quanzhou, China

**Keywords:** variety-seeking, theoretical perspective, underlying mechanism, measurement methods, consumption

## Abstract

Variety-seeking is a popular choice strategy in consumers’ daily lives, and many factors influence it. This study conducted a narrative and structured literature review based on three popular online academic databases to understand how researchers used influencing factors, adopted theoretical perspectives and underlying mechanisms, and developed measure methods in their studies. This paper consolidated and analyzed 61 articles on variety-seeking behaviors in consumer research, including empirical studies spanning from 2000 to 2021. This paper primarily focused on articles published at top tiers in the marketing literature. From these articles, a collection of internal and external factors, theoretical perspectives, underlying mechanisms, and measure methods adopted was summarized and tabulated for easy reference and comprehension. A research framework was developed to illustrate the relationships between influence factors and variety-seeking proposed by previous researchers. The literature review may not be exhaustive because variety-seeking behaviors could involve various research topics; however, the proposed research framework and suggested directions may be representative references for future research. This study is a more comprehensive literature review of variety-seeking behaviors in consumption research after 2000, and it contributes to a better understanding of the causes and effects of variety-seeking behaviors in consumption.

## Introduction

In daily life, when consumers face various selectable products, although they can repeatedly select their favorite products, they often choose ones in different categories, regarded as variety-seeking behavior (Kahn and Louie, 1990). To meet consumers’ needs and maximize their satisfaction (Sevilla et al., 2019), enterprises need to pursue the most accurate marketing segments. Consumption-related variety-seeking behavior provides an effective market segmentation standard for enterprises (Trivedi, 1999). In addition, such behavior helps increase sales volume and market share (Simonson and Winer, 1992), classify products, and effectively combine marketing strategies (Sela et al., 2019).

Variety-seeking behavior in consumption refers to individuals switching among products, categories, or brands to avoid the decreasing utility due to repeat purchases or consumption of the same products (Ratner et al., 1999). Over time, people tend to switch between options or select different options within a choice set (Shaddy et al., 2021). In the marketing domain, variety-seeking behavior also covers switching between marketing activities and services. Previous research found that consumers buy a certain number of diversified products even if they can repeatedly buy their favorite products from a given selection set (Ratner and Kahn, 2002). Repeating purchase or consumption reduces products’ marginal utility, thus reducing product attractiveness and causing boredom among consumers (McAlister, 1982; McAlister and Pessemier, 1982); existing products no longer meet consumers’ needs for stimulation (Choi, 1991). Therefore, consumers pursue freshness, change, and diversity by experiencing goods with different attributes to form satiety (Seetharaman and Che, 2009; Sevilla et al., 2016). This tendency shows that variety-seeking is common among consumers making product purchase decisions (McAlister, 1982) and a common choice strategy (Drolet and He, 2010).

Research on variety-seeking behaviors has a long history. Previous researchers have conducted valuable reviews on variety-seeking (McAlister and Pessemier, 1982; Kahn, 1995; Herrmann and Heitmann, 2006). However, the first two were published two or three decades ago. McAlister and Pessemier (1982) focused on the taxonomy of varied behavior and divided variety-seeking behaviors into two classes (decried and direct). Kahn (1995) similarly discussed three primary motivations for variety-seeking in the marketing literature: satiation/stimulation, external situation, and future preference uncertainty. The last one, Herrmann and Heitmann (2006), highlights the relevant literature on the domains of cultural psychology as well as marketing psychology with a review of consumers’ perception of variety-seeking. This study differs from the extant literature on the timeframe, method, and analysis. This study’s value lies in its narrative literature review on marketing and consumption articles published from 2000 to 2021 and their proposed conceptual models and frameworks. In contrast to previous reviews, this paper overviews the methodology approach, influencing factors, theoretical perspective, and underlying mechanism of variety-seeking behaviors in consumption. Based on these findings, a research framework of variety-seeking behaviors in consumption was developed to illustrate the inter-relationships among the adopted research constructs. This framework can provide a reference for researchers, serve as a research road map, and stimulate new ideas in future research in this subject area.

**Figure 1:**
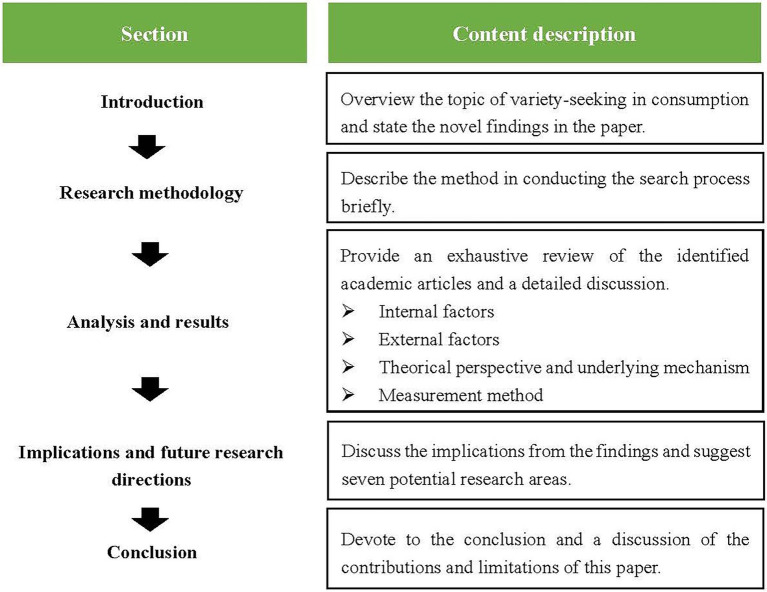
Flow diagram of sections.

This review article is organized as follows. This paper first briefly describes the method of conducting the search process. Next, this paper summarizes and discusses the internal and external factors of variety-seeking behaviors in consumption, followed by a generalization of the theoretical perspectives and underlying mechanisms of variety-seeking behaviors. Then, this paper reviews various measurement methods used by researchers and recommends the directions for future research based on the summarization of the current findings (Figure 1).

## Research Methodology

To investigate the work of previous researchers on variety-seeking behaviors in the consumption domain, this paper searched for empirical studies in the extant literature after 2000. The literature search was conducted from the Scopus database, which is the largest abstract and citation database of the peer-reviewed literature. The keyword “variety-seeking” was applied in the search process. The scope of this study is limited to the timeframe of 2000–2021 because there was only one literature review paper during this period. This search generated 293 records in total. Two hundred and thirty-five literature were omitted due to non-article type (8 records), neither SCI nor SSCI journal (70 records), non-English (2 records), specific subjects (e.g., children, older people, nonhumanity; 7 records), non-empirical paper (e.g., conceptual, review, and interview papers; 7 records), using modeling method (40 records), focusing on personality traits (52 records) and personal motivation (9 records) of variety-seeking, and no relation to consumption (40 records). Another 3 relevant papers were added. Finally, 61 papers were selected for in-depth analysis.

The search for relevant research in this process was by no means exhaustive; however, the findings nevertheless serve as a representative summary of the research conducted thus far. Only refereed journal articles were included in the study; conference papers, doctorate and master theses, textbooks, and documentaries were excluded because I believe refereed journal articles represent state-of-the-art research outputs (Chan and Ngai, 2011; Ngai et al., 2015). Moreover, because the current paper focus on “variety-seeking behavior in consumption,” most journals involving marketing and consumer psychology in top tiers were selected, such as *Journal of Marketing, Journal of Marketing Research, Journal of Consumer Research, Journal of Consumer Psychology, European Journal of Marketing, Marketing Letters, International Journal in Marketing, Journal of Consumer Psychology, and Journal of Personal and Social Psychology* as well as some journals in psychology and tourism and hospitality management. Finally, this study focused on papers presenting empirical studies, and the adopted variables and proposed models were reviewed and included in the framework.

## Analysis and Results

This section begins with a narrative review of the influencing factors adopted in the 61 identified empirical studies. The section then continues with the development of the research framework embedded in an analysis of theoretical perspectives and underlying mechanism, and measuring methods investigated by previous researchers in the formation of their conceptual models or frameworks. It should be noted that there are seven classical articles in the area published before 2000 described in this section, which are severed as background information.

### Internal Factors

The extant literature on variety-seeking behaviors in consumption considers five aspects of internal influencing: individual demographics, personality characteristics, emotional and physical states, sensory clues, and mindset.

#### Individual Demographics

A factor that could affect consumers’ variety-seeking behaviors is individual demographics, such as gender and age. For the effect of gender on variety-seeking behavior, researchers focused on the feminine menstrual cycle and gender differences. For example, across the reward domains of mating and hedonic food, Faraji-Rad et al. (2013) showed that women seek more variety in rewards when they are closer to ovulation because of their increased reward sensitivity caused by hormonal shifts during the fertile phase of the menstrual cycle. Similarly, Durante and Arsena (2015) revealed that women select a greater number of unique options from consumer product sets at high fertility, which is particularly strong for those in committed relationships. Chen et al. (2016) focused on two genders and demonstrated that men’s variety-seeking behavior in the product consumption domain increases in the presence of short-term, not long-term mating cues; by contrast, women’s variety-seeking behavior decreases in the presence of long-term but not short-term mating cues. For the effect of age on variety-seeking behavior, Novak and Mather (2007) found that younger adults selected similar levels of variety when choosing between what to consume immediately and later. By contrast, older adults consistently selected less variety when choosing something to be consumed later than immediately.

#### Personality Characteristics

Individual characteristics could influence consumers’ variety-seeking behaviors. Consumers who feel powerful (Jiang et al., 2014) are chronically indecisive (Jeong et al., 2016; Jeong and Drolet, 2016) and are novices (Sela et al., 2019) present more variety-seeking behaviors. First, building on an action-orientation perspective of power, Jiang et al. (2014) demonstrated that because high power is associated with a readiness to act and switching behavior generally requires taking actions in some form, consumers who feel powerful are more likely to switch in choice tasks. Second, Jeong et al. (2016) and Jeong and Drolet (2016) highlighted that chronic indecisiveness is associated with increased variety-seeking behavior. Chronically indecisive consumers (vs. not) feel less anxious and more positive after selecting a mix of products. Finally, consumers can acquire knowledge and signal their status in the marketplace during variety-seeking. Sela et al. (2019) argued that novices (vs. experts) perceive greater (vs. less) variety-seeking to indicate expertise because of perceived category breadth knowledge (vs. within-category discernment). Thus, novices (vs. experts) seek more (vs. less) variety to signal expertise. However, privately self-aware consumers are less inclined to opt for a varied choice set (Goukens et al., 2009).

In recent years, researchers explored luck beliefs, mindset traits, and self-oriented perfectionism in consumers’ variety-seeking behaviors. For instance, Zhao et al. (2021b) analyzed data from 593 respondents and showed that personal luckiness and belief in luck positively affect variety seeking. Li and Sun (2021) investigated 364 participants in the United States and found that consumers with a growth (vs. fixed) mindset are more likely to engage in variety seeking. As a purchasing strategy, variety-seeking also could be positively influenced by self-oriented perfectionism (Fu et al., 2021; *N* = 312).

Other personality traits, such as goal orientation and trait anger, influence variety-seeking behaviors depending on the situations. Considering decision tasks, Wu and Kao (2011) found that in the sequential choices for sequential consumption conditions, promotion-focused consumers tend to select a greater variety of items than prevention-focused consumers. The effect reversed in the simultaneous choices for sequential consumption conditions for prevention-focused consumers. Considering state anger, Zhao et al. (2021a) showed that people from relatively resource-abundant environments generally tend to seek variety when they are temporarily in an angry mood, independent of trait anger; although those with low trait anger tend to choose more variety compared to those with high trait anger. For people growing up in relatively resource-scarce environments, those with a low trait of anger tend to choose less variety when they feel angry than those with a high trait of anger.

#### Emotion and Physical State

Early researchers mainly explored the relationship between broad emotions (positive and negative feelings) and variety-seeking. For example, Kahn and Isen (1993) explored the influence of the positive effect on variety-seeking among safe and enjoyable products. The findings revealed that the positive affect induced by a gift bag of candy or sugarless gum enhanced consumers’ variety-seeking in choice behavior in three food categories (i.e., crackers, soup, and snack food) when circumstances did not make negative features of the items. However, the different degrees of positive feelings could produce distinct effects. Roehm and Roehm (2005) believed that more extreme positive moods might reduce variety-seeking—unlike mild positive moods—because the moderate stimulation obtained from variety-seeking is insufficient to meet people’s demands of extreme positive moods. The results of two pilot studies and two experiments showed that participants who viewed an ad cultivating an extremely positive mood switched less between candy bar snack brands on successive choices and selected fewer brands.

Then, researchers discussed how specific emotions and physical conditions, including positive and negative emotions (Chuang et al., 2008), sadness and happiness (Lin and Lin, 2009; Chien-Huang and Hung-Chou, 2010, 2012; Lin et al., 2011; Lin, 2014), local optimism and pessimism (Yang and Urminsky, 2015), and winning-losing perception (Chang et al., 2021), affect consumers’ decision-making behaviors when faced with multiple choices. In these moods and states, seeking variety helps people change their current status. For example, a study with 124 subjects demonstrated that people are likely to include more variety in their consumption decisions when they are induced to a negative emotion than a positive emotion (Chuang et al., 2008). Moreover, a series of research discussed the effect of two specific emotional states (sadness and happiness) on variety-seeking behaviors and found similar conclusions (Lin and Lin, 2009; Chien-Huang and Hung-Chou, 2010, 2012; Lin et al., 2011; Lin, 2014). These studies used choice task scenarios and revealed that participants with a sad mood selected more variety than those with a happy mood. Furthermore, Yang and Urminsky (2015) demonstrated that local optimism increases sequential choice consistency, whereas local pessimism increases sequential variety-seeking. Finally, Chang et al. (2021) found that consumers who have failed in a competition or not achieved a goal tend to seek less variety in their later consumption than consumers who have succeeded because losing feedback weakens consumers’ perception of their control of personal mastery.

Interestingly, some special physiological states have effects on variety-seeking, such as hunger (Goukens et al., 2007) and sleepiness (Huang et al., 2019). When people felt hunger or thirst, visual food or drink cues encouraged them to seek variety in relevant domains because these cues were more attractive to consumers who were in hunger or had just finished a fitness (Goukens et al., 2007). Another physiological state influencing variety-seeking is sleepiness. Huang et al. (2019) used multiple methods and revealed that sleepier consumers tended to seek more variety because of the need for arousal to maintain wakefulness. Particularly in Study1, a natural experiment based on the change of DST policy provided practical evidence for the positive effect of DST (decreasing short-term sleeping time and increasing sleepiness) on variety-seeking in products purchased by using Nielsen panel data (approximately 60,000 U.S. households data).

#### Sensory Clues

Individuals’ perception of the external circumstances depends on their keen sensory system, which receives various stimuli from the outside and then influences individuals’ mindset and decision making. People seek various choices when consuming to satisfy the sensory demand of vision (Maimaran and Wheeler, 2008; Deng et al., 2016; Huang and Kwong, 2016) and taste (Inman, 2001; Mukherjee et al., 2017), which have been discussed more in the current research.

Initially, the structural and superficial features of vision affect consumers’ variety-seeking behaviors subconsciously. First, individuals’ choices could be causally influenced by novel visual stimuli. For example, Maimaran and Wheeler (2008) demonstrated that exposure to variety arrays (arrays of differing shapes) increases variety-seeking, whereas exposure to uniqueness arrays (e.g., one circle among six squares) increases the choice of unique over common objects. Second, the display of products further influences variety-seeking in consumption because of the direction match between displays and eye movements. For example, Deng et al. (2016) used multiple methods (e.g., field study, laboratory study, and eye-tracking study) and demonstrated that consumers chose more variety (i.e., distinct fragrances, different candies, unique chocolates, and different types of lollipops) when alternatives were horizontally assorted or displayed. Third, a superficial feature can affect various perceptions even when the actual content or structure of an assortment remains unchanged. Huang and Kwong (2016) revealed that when the menu or catalog of an assortment is more difficult to read, the individuals perceived a higher variety. This readability effect stems from the subjective interpretation of the feeling of difficulty, that is, consumers generally endorse a lay belief that it is more difficult to make choices when they face a greater variety of options.

Subsequently, people might seek variety of taste stimuli to satisfy their needs. Inman (2001) believed that people switch more on sensory attributes (e.g., flavor) than nonsensory attributes (e.g., brand) to seek more pleasure. Inman (2001) used ACNielsen wand panel data for purchases of tortilla chips and cake mixes from almost 2000 consumers over 3 years (Study 1) and examined actual consumption behavior using a six-week consumption diary panel from over 850 consumers in two cities (Study 2) and employed a survey methodology (Study 3; 1056 responses) to verify his hypotheses: the difference of variety-seeking based on sensory and brand could be explained by “sensory-specific satiety,” that is, because of the high correlation between sensory-specific satiety and variety-seeking on sensory attributes, consumers switched more on flavors than brands. The research of Inman (2001) on sensory is broad, and subsequently, Mukherjee et al. (2017) discussed the relationship between a more specific taste—spicy and variety-seeking consumption. Based on embodied cognition and the metaphor “variety is the spice of life,” the authors found that spicy gustatory sensations (e.g., spicy vs. mild potato chips) activate a desire to be interesting that leads to greater variety in the subsequent unrelated choices (e.g., candy bars).

Ultimately, Lee and Sergueeva (2017) demonstrated an interesting “chewing effect” and argued that chewing more increases the viewing time and consumers’ thought-engagement while shopping and then increases variety-seeking behavior among consumers.

#### Mindset

Variety-seeking could also be the behavioral result of spontaneous thinking. The priming mindset influences variety-seeking in follow-up consumption, including past experiencing priming (Shen and Wyer, 2010) and semantic concept priming (Fishbach et al., 2011; Huang and Wyer, 2015; Zhang and Guo, 2019).

First, people’s past experiences can affect variety-seeking in the future. When individuals’ past behaviors associated with “same” were primed, they would get the feeling of boredom and then switch to a “different” decision rule (e.g., various types of herbal tea for four consecutive days) when performing a later task to eliminate this negative feeling (Shen and Wyer, 2010).

Second, the influence of semantic concepts on variety-seeking is nonconscious. For example, Fishbach et al. (2011) showed that when the negative concept related to “repetition” (e.g., boredom) was primed, it triggered an individual’s consumption structure based on satisfaction, that is, encouraging them to seek variety in order selection (e.g., buying smaller bottles of different shampoo, preferring CDs from different artists, staying in different hotels in the same city, visiting different cities in Europe, shopping at different stores, and choosing different snacks). Moreover, the influence of semantics is not only manifested in words related to choice behaviors but also has the same effect in words unrelated to choice behavior. For instance, Huang and Wyer (2015) found two opposite effects of mortality on variety-seeking: anxiety-inducing and concept-activation effects. The former was driven by the desire for stability and decreased the variety of individuals’ choices in an unrelated multiple-choice decision situation, whereas the latter induced a global processing style and increased variety-seeking. In addition, individuals’ temporal perspectives also trigger different seeking mindsets and affect variety-seeking behavior. Zhang and Guo (2019) demonstrated that past thinking brings familiar seeking and decreases variety-seeking, whereas future thinking induces novelty seeking and increases variety-seeking.

### External Factors

Whether or not people seek variety in the choice and decision-making process of consumption is not only affected by internal factors but also external environmental factors. These external environmental factors include social environment, physical environment, and marketing strategies.

#### Social Environment

The social environment’s influence on people’s daily behavior is subtle and has potential that is not easy to detect. Social factors that influence consumption variety are mainly from the two aspects of social relationships and social culture.

People would like to make various decisions to maintain well social relationships. The first social relationship comes from social pressure. Ratner and Kahn (2002) demonstrated that people choose more variety when they make decisions in public than in private because they expect to receive positive evaluations from others (perceived as “social pressure”; Ratner and Kahn, 2002). The second social relationship comes from interpersonal motivation. According to Ratner and Kahn (2002), Choi et al. (2006) showed that people have a stronger tendency to seek variety when they make choices for others. The explanations are as follows: (a) people should be responsible for their choices (the interpersonal mechanism; b) people expect to be satisfied more quickly when they choose for others (the intrapersonal mechanism). In addition, to maintain the self and interpersonal relationships, individuals’ perceived relational threat affects variety seeking in snack choices. Across three studies, Finkelstein et al. (2019) experimentally manipulated relational self-threat and found that those who experience high (vs. low) threat seek less variety, even when the same choice set is construed as having more (vs. less) variety. The third social relationship comes from the acquisition of interpersonal resources, that is, social influence. Ariely and Levav (2000) showed that the original groups choose more varied dishes than created groups, which is attributable to the interaction among group members and help individual satisfy goals of information gathering and self-presentation in the form of uniqueness in the group context. Chuang et al. (2013) maintained that to derive more enjoyment from a shared product, people show less variety and make choices consistent with the opinions of others in online information.

Furthermore, people in love form a special social relationship. For example, Etkin (2016) argued that consumers prefer more variety for joint consumption with their partners (e.g., going out to dinner, a movie, and a concert on a weekend), when they perceive more (vs. less) time ahead in a committed relationship. Huang and Dong (2019) found that a salient relationship state—romantic crush—can increase consumers’ variety-seeking tendency in unrelated consumption situations.

Variety-seeking behaviors in consumption could be influenced by the root of social culture. Kim and Drolet (2003) highlighted that as a choice rule, people in a unique culture display greater variety. Similarly, Yoon et al. (2011) reported that because members of a collectivist culture tend to follow group members’ choices, their choices in snacks are associated with a higher uniformity-seeking tendency than those of individualistic cultural backgrounds. Moreover, building on the compensation consumption literature, Yoon and Kim (2018) demonstrated that consumers with low socioeconomic status and perceive low economic mobility (e.g., economically stuck consumers) seek more variety than others to compensate for their lack of personal control. Finally, political ideology has a counterintuitive effect on variety-seeking. Fernandes and Mandel (2014) showed that conservatism is positively related to variety-seeking because of social normative concerns.

#### Physical Environment

The physical environment factors that affect variety-seeking in consumption mainly include the space environment and time point.

First, the constraints of a physical space enhance variety-seeking in consumption. Based on resistance theory, Levav and Zhu (2009) found that consumers confined by space make more various and unique choices to resist the invasion of their private space and seek freedom. The authors revealed that people in narrower aisles sought more varied candies than people in wider aisles (Study 1), and this effect of confinement in narrow aisles is extended to more unique choices in charities (Study 2), particularly in those with high chronic reactance tendency (Study 3). Moreover, the field study (94,110,967 usable transactions) used crowding as a proxy for confinement and found a positive relationship between crowding and variety-seeking in real grocery purchases.

As another type of space environment, the restaurant atmosphere, store environment, and web feature also could influence consumers’ variety-seeking. For example, Ha and Jang (2013a) collected 309 useable responses and pointed out that consumers’ desired hedonic and utilitarian values of the restaurant positively influence their variety-seeking intentions. Similarly, according to 617 usable responses to the restaurant experience, Ha and Jang (2013b) showed that atmospheric quality, overall boredom, and boredom with atmospheric attributes significantly influence dinners’ variety-seeking intentions positively. For the off-line store environment, Mohan et al. (2012) investigated 350 shoppers in Dubai and established that the store environment (including lighting, scent, and music) affects variety-seeking positively. For the online web feature, with 698 usable responses, Hung et al. (2011) demonstrated that quality web features affect interpersonal trust and platform credibility positively, and both constructs drive a user’s online community usage and brand variety-seeking behavior.

Second, the objective time of day could further influence variety-seeking in consumption. Given the influence of physical laws, people exhibit different levels of variety-seeking in consumption at different time points. For example, Roehm and Roehm (2004) found that people are more likely to seek variety in candy choices at low arousal (e.g., 9 AM; 10:00 AM–11:20 AM) than peak arousal (e.g., 4 PM; 3:10 PM–4:20 PM) moments of the day. However, the latest research provided an inconsistent result of diurnal variation in variety-seeking. Based on circadian rhythms in chronobiology, Gullo et al. (2019) applied four studies, including an empirical analysis of millions of purchases, and stated that individuals pick less varied flavors of yogurt when choosing in the morning. Furthermore, different external environments and changes in life events can change people’s variety-seeking. Koschate-Fischer et al. (2018) showed that consumers reduce their variety-seeking tendency after experiencing a life event (1,475 panelists).

#### Marketing Strategy

The marketing strategy influences consumers’ variety-seeking behaviors primarily in the purchase stage. Kahn and Louie (1990) first studied the relationship between retail stores’ promotion strategy and variety-seeking. They found that if only one shampoo brand is promoted and people are generally loyal to the last brand purchased, they tend to switch among shampoo brands when the promotion is withdrawn.

In the later stages, the research on the impact of marketing strategy has become in-depth, such as product packaging, product bundle strategy, product category and information, and product assortment. For example, product packaging uniformity is associated with arousal potential and influences consumers’ variety-seeking. Roehm and Roehm (2012) showed that consumers’ variety-seeking is greater in product categories where packaging is similar among competitors.

Furthermore, the product bundle strategy affects consumers’ variety-seeking when they experience multiple products. Mittelman et al. (2014) found that consumers seek more variety when choosing from single offerings (e.g., a choice of two individual candy bars) than from bundled offerings (e.g., a choice of a bundle of two candy bars), which is termed “offer framing effect.” Kim et al. (2018) based on the decision-framing effect and found that travelers show higher variety-seeking in travel package decisions when the bundle package is selected from a combined decision rather than from two single decisions.

Moreover, product category and product information affect variety-seeking behavior. For the product category, several researches were conducted from various perspectives. Based on a specific-abstract categorization strategy, Kim and Yoon (2016) showed the “category specificity effect” and revealed that individuals are likely to order a greater variety of dishes when the menu contains no category labels or abstract category labels due to the enhanced perception of variety offered in the menu. Baltas et al. (2017) indicated that in hedonic product categories, consumers seek more variety in sensory attributes, whereas, in utilitarian product categories, they seek more variety in functional attributes. What is the difference between digital and consumable goods? Adomavicius et al. (2015) showed a reduction in behavioral effects of bundle cohesion and timing on variety of preferences for digital goods. For the product information, Lin et al. (2017) indicated that when people purchase products for themselves, the presence of risky information and health claims, and high product involvement promote more variety-seeking.

Finally, as detailed in Section “Theoretical Perspective and Underlying Mechanism”, the displays and assortments of products affect consumers’ variety-seeking behaviors. For example, the display of novel geometric figure arrangement combinations (various shapes) increases consumers’ variety-seeking (Maimaran and Wheeler, 2008). The horizontal assortment is easier to process and can increase individuals’ perceived variety, thereby ultimately leading to greater variety-seeking (Deng et al., 2016; Table 1).

**Table 1:** Factors investigated in variety-seeking bahavior in consumption.

**Factors**	**References**	***N***
Internal factor		
1. Individual demographics		4
• Gender	Faraji-Rad et al., 2013, Durante and Arsena, 2015, and Chen et al., 2016	
• Age	Novak and Mather, 2007	
2. Personality characteristics		10
• Self-awareness	Goukens et al., (2009)	
• Goal orientation	Wu and Kao, 2011	
• Power	Jiang et al., 2014	
• Choroically indecisiveness	Jeong and Drolet, 2016 and Jeong et al., 2016	
• Knowledge	Sela et al., 2019	
• Luck believes	Zhao et al., 2021b	
• Mindset traits	Li and Sun, 2021	
• Self-oriented perfecionism	Fu et al., 2021	
• Trait anger	Zhao et al., 2021a	
3. Emotion and physical state		11
• Emotion	Roehm and Roehm, 2005, Chuang et al., 2008, Lin and Lin, 2009, Chien-Huang and Hung-Chou, 2010, 2012, Lin et al., 2011, Lin, 2014, Yang and Urminsky, 2015, and Chang et al., 2021	
• Physical state	Goukens et al. (2007) and Huang et al. (2019)	
4. Sensory clue		6
• Vision	Maimaran and Wheeler (2008), Deng et al. (2016), and Huang and Kwong (2016)	
• Taste	Inman, 2001 and Mukherjee et al., 2017	
• Chewing	Lee and Sergueeva, 2017	
5. Mindset		4
• Experience priming	Shen and Wyer, 2010	
• Semantic concept	Fishbach et al., 2011, Huang and Wyer, 2015, and Zhang and Guo, 2019	
External factor		
1. Social environment		11
• Social relationship	Ariely and Levav, 2000, Ratner and Kahn, 2002, Choi et al., 2006, Chuang et al., 2013, Etkin, 2016, Huang and Dong, 2019, and Finkelstein et al., 2019	
• Social culture	Kim and Drolet, 2003, Yoon et al., 2011, Yoon and Kim, 2018, and Fernandes and Mandel, 2014	
2. Physical environment		8
• Space and atomsphere	Levav and Zhu, 2009, Hung et al., 2011, Mohan et al., 2012, and Ha and Jang, 2013a,b	
• Time	Roehm and Roehm, 2004, Gullo et al., 2019, and Koschate-Fischer et al., 2018	
3. Marketing strategy		9
• Product package	Roehm and Roehm, 2012	
• Product bundle	Mittelman et al., 2014 and Kim et al., 2018	
• Product category and information	Adomavicius et al., 2015, Kim and Yoon, 2016, Baltas et al., 2017, and Lin et al., 2017	
• Assortment	Maimaran and Wheeler, 2008 and Deng et al., 2016	
Total		63

*Based on the previous research of variety-seeking behavior in consumption. Two are duplicated due to research desgin: Maimaran and Wheeler (2008), Deng et al. (2016)*.

### Theoretical Perspective and Underlying Mechanism

Many theories and effects are used in the extent of variety-seeking behaviors in consumption research to explain the underlying mechanism that consumers seek variety during decision making and purchasing. The theoretical perspectives and underlying mechanism can be summarized in six groups: optimal stimulus level, personality characteristics perspective, emotional coping perspective, compensatory consumption perspective, environmental psychology perspective, and evolutionary psychology perspective. Several significant theories and effects were selected in each group and briefly discussed.

#### Optimal Stimulus Level

*Optimal stimulation level theory* is an early and fundamental theory to explore variety-seeking behavior in consumption, which is widely applied in the existing literature. One reason consumers seek variety in product selection is to meet their demand for stimulation (Menon and Kahn, 1995). According to *optimal stimulus level theory*, the relationship among internal individual factors, external environmental factors, and consumer preference response can be represented by an inverted U-shaped curve function. In this curve function, the peak vertex of the curve is the optimal stimulus level, the attribute set under this level can cause the consumer’s satisfaction to reach the highest level, and the stimulus level on both sides of the vertex is too low or too high to satisfy the consumer (McAlister, 1982). If consumers often buy the same product or category, their effective stimulus level in decision-making decreases. Therefore, to obtain greater stimulation, consumers attempt to buy different products or products to achieve their goals (Roehm and Roehm, 2005). In addition, because of physiological stimulation and arousal (e.g., body temperature), consumers receive the least stimulation in the morning and produce a lower variety-seeking (Gullo et al., 2019). *Arouse theory* was also applied by Roehm and Roehm (2004) and Huang et al. (2019) to explain consumers’ need for stimulation.

#### Personality Characteristics

As the internal influencing factors, much research focus on the effect of personality characteristics on variety-seeking behavior in consumption from the individual perspective. As a result, theories and underlying mechanism of these effects are in varied forms, which are mostly based on the consumers’ personality traits. For example, *Self-awareness theory* and *Goal orientation theory* were adopted to explore how consumers’ self-awareness and promotion–prevention orientation affect their variety-seeking behaviors (Goukens et al., 2009; Wu and Kao, 2011). According to *Implicit Theory*, consumers with a growth (vs. fixed) mindset are more likely to engage in variety seeking due to their changing preferences (Li and Sun, 2021). Based on *Signal theory*, Sela et al. (2019) found that variety-seeking behavior can serve as a signal to indicate expertise. Personality characteristics also can shape consumers’ *variety-seeking mindset* and then promote variety-seeking behaviors (Kim and Yoon, 2016; Zhang and Guo, 2019).

#### Emotional Coping

Emotions are the psychological states that people need to face every day. Different emotional states bring different stimulation levels to consumers. Based on the *Mood evaluation framework*, compared with positive emotions (such as happiness), negative emotions (such as sadness) bring low satisfaction to consumers; therefore, consumers experiencing negative emotions increase their satisfaction through variety-seeking behaviors (Roehm and Roehm, 2004; Lin and Lin, 2009; Chien-Huang and Hung-Chou, 2010, 2012; Lin et al., 2011; Lin, 2014). Building on *Processing style theory*, mortality salience increases variety-seeking behaviors in consumption by influencing an individual’s global processing style (Huang and Wyer, 2015). Variety-seeking behavior in consumption is observed to help cope with and alleviate the negative effects of negative emotions. *Optimal stimulus level theory* also can help explain this. In an extremely positive mood state, consumers reduce their variety-seeking behaviors because the stimulus provided by variety-seeking behaviors in consumption belongs to the middle level, which is not enough to meet the demand for extreme positive emotions for stimulation (Roehm and Roehm, 2005). However, consumers’ variety-seeking behavior when in a mildly positive mood (moderate degree) is influenced by product characteristics, such as security and pleasantness (Roehm and Roehm, 2005).

#### Compensatory Consumption

*The theory of sense of control* is the core element in the compensatory consumption perspective. Compensatory consumption means that consumers engage in certain consumption behaviors to make up for the lack of psychological needs because of the lack of overall self-esteem or self-realization (Gronmo, 1988). The essential feature of compensatory consumption is to make up for psychological defects or threats through consumption behavior, emphasizing consumption behavior as an alternative means and tool—rather than functional value—to meet demand. Compensatory consumption is a kind of pure psychological consumption and self-presentation of psychological imbalance. Therefore, in a variety of scenarios in which psychological defects and threats might occur, variety-seeking in consumption can be used as an alternative means to meet psychological needs and cope with threats. For example, because consumers with low social status and perceived low social mobility tend to have a low sense of personal control, they show more variety-seeking behaviors in consumption to compensate for their psychological defects (Yoon and Kim, 2018). If people in love are “left out,” their sense of control in a romantic relationship is reduced—to restore a sense of control, they seek a variety of choices in consumption (Huang and Dong, 2019).

#### Environmental Psychology

As mentioned earlier, environmental psychology focuses on the relationship between the environment and individuals’ psychology and behavior. The environment includes the physical and social environments, both of which have an important impact on people’s behavior.

First, spatial perception is a physical environment. According to the *Resistance theory*, if consumers feel constrained (such as in narrow aisles and among crowded people), they resist the invasion of private space through more various and unique choices, which is equivalent to resisting the constraint (Levav and Zhu, 2009). In addition, according to *the spontaneous effect*, a diversified display of commodities stimulates consumers’ variety mindset, leading to the emergence of variety-seeking in consumption (Maimaran and Wheeler, 2008). Finally, because of a match between the human binocular vision field and the dominant direction of eye movements (which are both horizontal in direction), it is easier for horizontal (vs. vertical) displays to be processed. This *processing fluency* allows people to browse information more efficiently, which increases perceived assortment variety and ultimately leads to more variety being chosen (Deng et al., 2016).

Second, social groups and the cultural and political factors in the social environment affect variety-seeking behaviors in consumption from different aspects. The influence of society on consumer behavior is mainly constrained by social norms, which could be generalized by *interpersonal and intrapersonal motivation*. To maintain consistency with the group (normative constraints) and given the influence of group norms or opinion leaders, people might change their original consumption habits that are inconsistent with the reference group (to promote variety-seeking in consumption) or insist that the original consumption habits are consistent with the group (to prevent variety-seeking in consumption; Ariely and Levav, 2000; Ratner and Kahn, 2002; Choi et al., 2006; Fernandes and Mandel, 2014; Etkin, 2016; Finkelstein et al., 2019). *Cross-culture theory* explains the individual difference in variety-seeking from the root cultural perspective, and collectivism vs. individualism is the main cultural difference. Members of a collectivist culture tend to consist of group members, and their choices are associated with a less variety-seeking tendency than those of individualistic cultural backgrounds (Kim and Drolet, 2003; Yoon et al., 2011).

#### Evolutionary Psychology

Evolutionary psychology research focuses on the influence of women’s ovulation period and gender differences, and scholars use *the carry-over effect* to investigate variety-seeking behavior in consumption between men and women. Given the influence of hormonal changes during the physiological cycle and to meet reproduction needs, women may be more sensitive to rewards and seek variety when seeking a spouse; therefore, they seek various and novel choices extend to irrelevant consumption choice tasks (Faraji-Rad et al., 2013; Durante and Arsena, 2015; Chen et al., 2016). From an evolutionary perspective, *Life-history theory* demonstrates that people from relatively resource-abundant or relatively resource-scarce childhoods (i.e., childhood SES) often respond differently when faced with an environmental threat (Griskevicius et al., 2013). Variety-seeking may be a risk reduction strategy against uncertainty about future taste preferences in simultaneous choices for future sequential consumptions among people from different degrees of resource childhoods (Zhao et al., 2021a; Table 2).

**Table 2:** Theories and underlying mechanism used in variery-seeking behavior in consumption.

Theories and underlying mechanism		References
**Optimal stimulus level**		
	Optimal stimulus level theory	Roehm and Roehm, 2005, Chuang et al., 2013, Ha and Jang, 2013a,b, and Gullo et al., 2019
	Arouse theory	Roehm and Roehm, 2004 and Huang et al., 2019
**Personality characteristics**		
	Goal systems theory	Goukens et al., 2007
	Self-awareness theory	Goukens et al., 2009
	Goal orientation theory	Wu and Kao, 2011
	Signal theory	Sela et al., 2019
	Implicit theory	Li and Sun, 2021
	Courage–ability–willingness theory	Zhao et al., 2021b
	Theroies of metaphors and embodied cognition	Mukherjee et al., 2017
	Theory of mental budgeting	Fu et al., 2021
	The framing effect	Lin et al., 2017, Kim et al., 2018
	The spontaneous effect	Shen and Wyer, 2010 and Fishbach et al., 2011
	The variety-seeking mindset	Kim and Yoon, 2016 and Zhang and Guo, 2019
**Emotional coping**		
	Emotion-maintenance theory	Chuang et al., 2008
	Processing style theory	Huang and Wyer, 2015
	Mood evaluation framework	Lin and Lin, 2009, Chien-Huang and Hung-Chou, 2010, 2012, Lin et al., 2011, and Lin, 2014
	Self consisitency	Yang and Urminsky, 2015
	Reduce uncertainty	Jeong and Drolet, 2016 and Jeong et al., 2016
**Compensatory consumption**		
	Theory of sense of control	Yoon and Kim, 2018, Huang and Dong, 2019, and Chang et al., 2021
**Evironmental psychology**		
	Resistance theory	Levav and Zhu, 2009
	Corss-culture theory (collectivism vs. individualism)	Kim and Drolet, 2003 and Yoon et al., 2011
	Source credibility framework	Hung et al., 2011
	The spontaneous effect	Maimaran and Wheeler, 2008
	The framing effect	Mittelman et al., 2014
	The carry-over effect	Huang and Kwong, 2016
	Interpersonal and intrapersonal motivation	Ariely and Levav, 2000, Ratner and Kahn, 2002, Choi et al., 2006, Fernandes and Mandel, 2014, Etkin, 2016, and Finkelstein et al., 2019
	Processing fluency	Deng et al., 2016
	Satiation	Baltas et al., 2017
	Positive affect	Mohan et al., 2012
**Evolutionary psychology**		
	The carry-over effect	Faraji-Rad et al., 2013, Durante and Arsena, 2015, and Chen et al., 2016
	Life-history theory	Zhao et al., 2021a
	Emotion regulation	Novak and Mather, 2007

*Based on the previous research of variety-seeking behavior in consumption*.

### Measurement Method

Presently, variety-seeking behavior in consumption could be measured by the survey and experimental methods. Although the diversified consumption scenarios and variety-seeking measurement methods used by scholars are different in research using the experimental method as the paradigm, they also can be roughly divided into three types: scenario simulation, real choices in experiments, and real shopping behavior data.

#### Measurement Scale

In the survey method, five-point and seven-point Likert scales are applied to measure participants’ variety-seeking. The items in the scales were adopted from the previous studies. Participants assess how much they would like to purchase or consider new and unfamiliar brands and products. To test the hypothesized relationships, structural equation modeling (SEM) is performed in research. For example, Hung et al. (2011) measured variety-seeking from Kahn et al. (1986), which used five-point Likert scales (1 = strongly disagree, 5 = strongly agree). In the research of Fu et al. (2021), variety seeking was measured by three items from Grünhagen et al. (2012), with a five-point probability scale ranging from 1 (not probable) to 5 (very probable). A sample item is “I am willing to see different food products and brands.” In the research of Zhao et al. (2021b), variety seeking was measured with the five-item Variety Seeking Scale (Helm and Landschulze, 2009). A sample item is “Buying the same product or brand is boring, even if the product or brand is good.” Furthermore, Van Trijp et al.’s (1996) seven-point Likert type scale is also used by Ha and Jang (2013a,b) and Liu et al. (2021). A sample item is “I am very cautious in trying new or different products.”

#### Scenario Simulation

In the experimental method, researchers usually describe a consumption scenario and ask participants to imagine a choice in this scenario. The two common choices are simultaneous selection (multiple products or services choices at one time) and sequential selection (one product or service at a time, multiple choices in a row). These choice scenarios include food consumption, purchasing behavior, tourism consumption, and so on. Typically, researchers use the number of products or services selected by participants as the variety-seeking index.

Purchasing and selecting products tasks are frequently used as selection scenarios in the research, the majority of which are used for the food selection task. For example, Simonson (1990) asked participants to imagine that they are going to the supermarket, and their shopping list contained eight products, each a different type of good. The author asked the participants to choose one good every day or choose for three days at a time (Simonson, 1990). Many studies followed this research design (Mukherjee et al., 2017; Gullo et al., 2019), such as the purchasing socks task (five out of nine; Yoon and Kim, 2018), the outing task (potato chips choose three out of four; Chen et al., 2016), the teatime reservation task (25 snacks, 20 options; Roehm and Roehm, 2005), and the sandwich pre-arranged task (seven out of nine; Goukens et al., 2007). The number of brand categories that participants selected is recorded as variety-seeking. In addition, the drinks choosing task is also applied in the research. For example, Goukens et al. (2007) designed a drink-selection scenario, in which participants imagined that they received a gift basket and could choose six drinks among eight flavors. Similarly, volunteer tasks (five out of six; Chen et al., 2016) and the tea beverage task (four options) exist (Shen and Wyer, 2010).

Some studies also adopted other forms of selection scenarios. For example, Levav and Zhu (2009) designed the charitable donation task, in which participants can donate their reward for participating in the experiment to one, several, or all six charities. Goukens et al. (2007) designed a holiday scenario, in which participants imagined that they had won a free trip to Sri Lanka, including air tickets, accommodations, and four experience activities. They could choose four out of 16 activities: four beach activities, four outdoor adventures, four sports activities, and four cultural experiences (Goukens et al., 2007, 2009; Huang and Wyer, 2015). Furthermore, other studies considered cross-product categories’ choice tasks, such as food and stationery categories (tea drinks, potato chips, and books; Shen and Wyer, 2010; Huang and Wyer, 2015), daily necessities categories (lipstick, high heels, yogurt, candy, nail polish, and restaurant; Durante and Arsena, 2015), and entertainment activities (drinks, movies, weekend activities; Etkin, 2016; Gullo et al., 2019). In addition, some studies also used behavior switching to measure the variety-seeking in consumption (Jiang et al., 2014). For example, Yang and Urminsky (2015) used magazines, music, and movies as experimental materials and measured their preference conversion through participants’ choices before and after.

#### Real Choice in the Experiment

This measurement method requires participants to make real choices during the experiment, but participants were not aware that their choices were influenced and recorded. This measurement method makes the variety-seeking behavior appear in a more realistic scenario, reflecting people’s relatively real, and potential choices and increasing the validity of the research results. Researchers usually let participants choose by selecting experimental rewards or compensation.

In the real selection task, many studies use candies or chocolate as the selection stimuli that are finally selected as rewards or compensation for participation considering the convenience of the experiment and the sample. For example, Simonson (1990) rewarded participants with snacks and asked them to choose between sweet and salty snacks (three total groups). Similarly, five rewards were available for choosing among nine snacks (Choi et al., 2006; Durante and Arsena, 2015), up to five desserts, candies, or yogurts (Yoon and Kim, 2018; Huang et al., 2019), the candy list selection task (Ratner and Kahn, 2002; Roehm and Roehm, 2004), choosing three out of six types of candies (Levav and Zhu, 2009), rewarding three of four lollipop flavors (Chen et al., 2016), and the chocolate selection design for three out of four choices and six choices (Maimaran and Wheeler, 2008; Yoon and Kim, 2018). In addition, Deng et al. (2016) fabricated a research purpose as investigating the influence of virtual store lighting on shopping patterns and gave each participant two dollars to buy the displayed candies.

In addition to snack choice tasks, researchers also asked participants to choose stationery frequently used by college student samples. For example, Levav and Zhu (2009) asked participants to choose three out of six color highlighters as rewards in Experiment 4, which was also applied in Gullo et al. (2019). Another distinct and interesting selection task was the flower arrangement task designed by Mittelman et al. (2014), who provided participants with differently colored roses that needed to be put in vases, and used the number of selected colors as a variety-seeking measure.

#### Real Purchase Behavior Data

In recent years, researchers began to call for the study of consumer behavior in the real environment. Scholars used purchasing data generated by consumers to measure the variety-seeking in consumption and analyzed variety-seeking using data obtained from various methods. Among them, consumer panel data from Nielsen and retail stores are often used by researchers (Inman, 2001; Levav and Zhu, 2009; Yoon et al., 2011; Gullo et al., 2019); in such research, researchers typically used the ratio of the number of categories purchased to the total number of categories as a variety-seeking measure. In addition, some researchers conducted field studies among cities (Koschate-Fischer et al., 2018), field experiments (Yoon et al., 2011; Deng et al., 2016), or natural experiments (Huang et al., 2019) to obtain real behavior data. Kahn et al. (1986) provided an analytical framework for how to use panel data to define and measure variety-seeking and offered seven simple and verifiable models commonly used in the marketing domain.

Universal Product Codes (UPCs) are useful and helpful when adopted to calculate consumers’ variety-seeking behaviors. Inman (2001) used UPCs to construct two indexes to measure consumers’ observed switching (the observable flavor or brand switching percentage) and expected switching (which is calculated based on Zero Order; Grover and Srinivasan, 1987). The switching index is then calculated as: Relative Switching Intensity = (Observed – Expected) _Flavor_–(Observed – Expected) _Brand_.

Levav and Zhu (2009) used purchasing data in Study 5 to compute a variety-seeking index that captured the extent of variation in a transaction. This was computed for each customer by dividing the number of unique UPCs purchased in a category by the category’s total purchases. The authors used its log odds to conduct an OLS regression using this variety index, with log (variety/(1- variety)), as the dependent variable. Gullo et al. (2019) followed Levav and Zhu (2009), using scanner panel data from a major grocery chain’s single California location. They defined variety as the number of unique UPCs purchased in a category relative to the number of total items purchased. Similarly, Huang et al. (2019) used the Chicago Nielsen consumer panel data set and the number of UPCs per trip to measure variety-seeking.

In addition, Koschate-Fischer et al. (2018) combined two datasets, an individual-level consumer panel and a survey, collected over 3 years. They used the change in SOW and SOU to compute variety-seeking. SOW is the share of wallet, defined as the percentage of money a customer allocates to the preferred brand in a category (our unit of analysis). SOU is the share of units, defined as the percentage of units purchased for the preferred brand in a particular category, controlling for price level effects (Table 3).

**Table 3:** Summary of measurements of variety-seeking behavior in consumption in literature.

**Measurement**	**Description**	**Scenairo**	**Example**	**References**
Scale	Participants assess how much they would like to purchase or consider new and unfamiliar brands and products.			Hung et al., 2011
		Ha and Jang, 2013a,b
		Liu et al., 2021
		Fu et al., 2021
		Zhao et al., 2021b
Scenario stimulation	Participants were prvided with a consumption scenario. They were also asked to image and make a choice in this situation.	Product purchase	Shopping list	Chen et al., 2016
				Mukherjee et al., 2017
				Gullo et al., 2019
				Chang et al., 2021
		Selection task	Donation	Levav and Zhu, 2009
			Holiday activity	Goukens et al., 2007
			Snacks choice	Goukens et al., 2009
				Chen et al., 2016
				Jeong et al., 2016
			Product selection cross categories	Shen and Wyer, 2010
				Durante and Arsena, 2015
				Huang and Wyer, 2015
Real choice in experiment	Participants made real choices during the experiment, but they did not realize that their choices had been influenced and recorded.	Real choice	Snacks choice	Choi et al., 2006
				Durante and Arsena, 2015
			Stationery selection	Levav and Zhu, 2009
				Gullo et al., 2019
			Flower arrangement task	Mittelman et al., 2014
Real shopping behavior data	Researchers measured variety-seeking behavior in consumption with the purchase data actually generated by consumers. Data can be obtained from different sources, such as datasets and survey.	Shopping experience in real world	Panel data	Inman, 2001
				Levav and Zhu, 2009
				Gullo et al., 2019
			Survey cross cities	Koschate-Fischer et al., 2018
			Field experiment	Deng et al., 2016
			Natural experiment	Huang et al., 2019

Based on the previous research of variety-seeking behavior in consumption.

## Implications and Future Research Directions

Based on the proposed research framework of variety-seeking behaviors in consumption, this section discusses the implications of the aforementioned findings and identifies opportunities for future research in variety-seeking.

### Implications of the Findings

**Figure 2:**
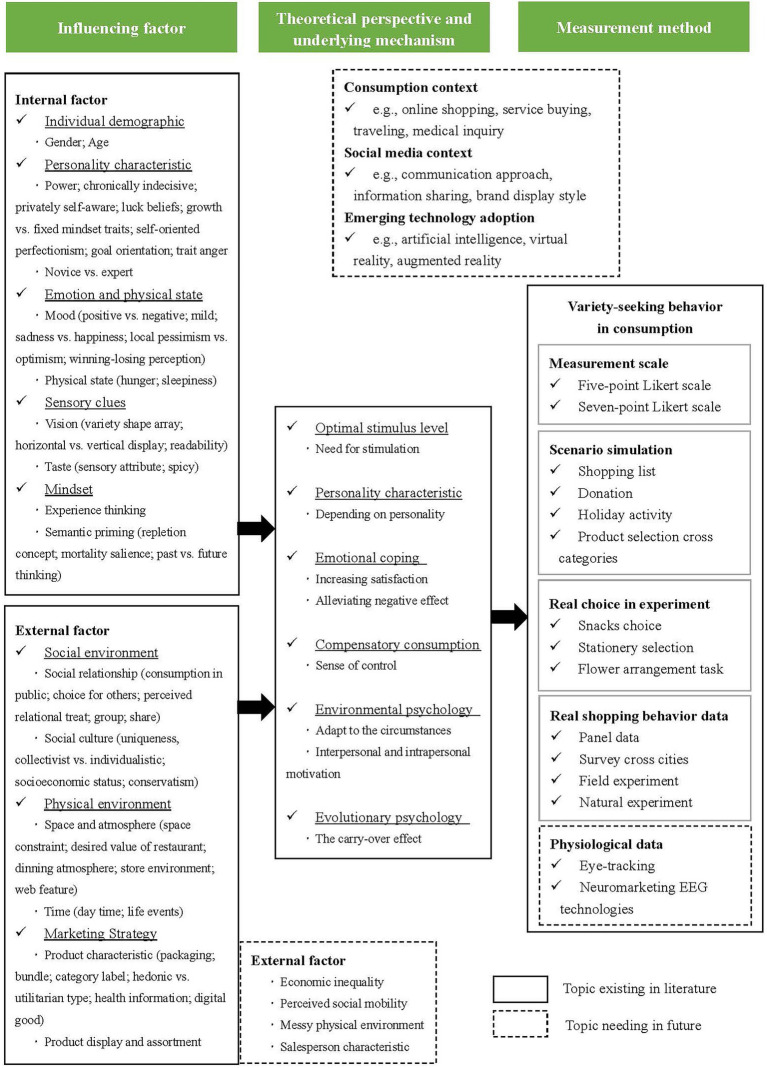
Research framework of variety-seeking behavior in consumption. Based on the previous research on variety-seeking behavior in consumption.

This literature review shows that numerous researchers have studied the relationships between various internal and external factors and variety-seeking behaviors from distinct theoretical perspectives by using various measurement methods. All these attributes are delineated in the proposed framework of variety-seeking behaviors in consumption (see Figure 2).

Concerning internal factors, gender and age in the category of individual demographics are the two most adopted aspects. Researchers have attempted to discover the different effects of variety-seeking between males and females and between younger and older people. Power, indecisiveness, and novice have attracted considerable research attention for personality characteristics. Some researchers also study the effects of various emotions, such as positive moods, sadness and happiness, local optimism and pessimism, and winning-losing perception. In addition, some notable and interesting physiological states, such as hunger and sleepiness, are discussed in variety-seeking behaviors in consumption. In the category of sensory clues, researchers have focused on investigating the effect of vision (e.g., novel visual stimulus, the display way of products, a superficial feature) and taste (e.g., flavor and spicy). A few papers examine how consumers’ mindset affects variety-seeking behaviors.

Regarding external factors, social relationships and social culture are widely used to investigate the effect of the macro social environment on variety-seeking, as variety-seeking behaviors could meet some social motivations. Space and temporal factors from the external environment can also influence variety-seeking in consumption. Moreover, some researchers are concerned with marketing strategies in variety-seeking, including product packaging, product category, attribute type, and the displays and assortments of products.

Among the theoretical perspectives and underlying mechanisms, optimal stimulus level theory is the most fundamental and widely applied theory to explain consumers’ variety-seeking behaviors when facing external stimuli. Personality characteristics perspective is applied in much research. How these traits affect variety-seeking depends on core characteristics of individual difference, which is mostly related to “changing” or “uniqueness.” Emotional coping is another common perspective used by researchers, and it has been explored from the board mood (e.g., positive mood) to the specific mood (e.g., happiness, sadness, mortality salience). Some researchers found that variety-seeking can meet the lack of psychological needs: the compensatory consumption perspective. In recent years, researchers have drawn on environmental psychology and evolutionary psychology theories to examine how environmental factors and gender differences affect consumers’ variety-seeking behaviors, which provides novel insights into the literature.

The four main measurement methods used by researchers include measurement scale, scenario simulation, real choices in experiments, and real shopping behavior data. The measurement scale is adopted from previous studies. Scenario simulation is applied primarily to the experiment method, and the number of consumers’ various choices is used as the variety-seeking index. Researchers also adopt real choices in experiments and real shopping behavior data from real retailers to investigate consumers’ variety-seeking behaviors, reflecting their actual choices and behaviors.

### Future Research Directions

This paper reviews and combs through the related research on variety-seeking behavior in consumption. The current framework summarizes internal and external influencing factors, theoretical perspective, and underlying mechanisms and measurement methods of variety-seeking behavior in consumption, which has theoretical value for further insights into the literature. Despite the ongoing progress, future research can focus on the following aspects.

First, additional research is needed to widely and deeply explore the external factors influencing consumption variety-seeking behavior. The proposed research framework shows that most past research concentrated on internal factors; thus, future research should extend to external environmental factors. Regarding the social environment, other factors, such as economic inequality (Goya-Tocchetto and Payne, 2022) and perceived social mobility (Wang et al., 2022), are also rooted in people’s lives and determine their thinking styles and behaviors; therefore, it should be determined how these societal factors drive the variety-seeking behavior in consumption. Regarding the physical environment, the space environment has many presentation modes. Excepting narrow space, individuals may also experience a chaotic physical environment (Vohs et al., 2013), encouraging them to break the tradition and change consumers’ preferences, choices, and behaviors. Future research could explore whether physical order in the external consumption environment influences variety-seeking behaviors. In terms of marketing strategy, the influence of salespersons has been little concerned. Many characteristics of salespersons affect consumers’ emotional or irrational decision-making and purchase intentions, such as appearance attractiveness (Li et al., 2021) and tone and voice (Liu et al., 2021). Future research could investigate variety-seeking behavior in consumption from the aspect of salespersons.

Second, future research could investigate variety-seeking behavior in consumption with specific situations, such as catastrophes and significant public health affairs. In these specific situations, variety-seeking behavior in consumption also shows particular functions. For example, consumers’ psychology and behavior have changed during the COVID-19 pandemic. Given that this period differs from previous times, the factors affecting consumers’ variety-seeking behavior should be determined, along with the psychological process and underlying mechanisms. From the perspective of compensatory consumption theory, it is also worth considering whether the health, economic, social, informational, and environmental threats caused by the epidemic can influence variety-seeking behavior in consumption (Campbell et al., 2020). These threats may decrease consumers’ perceived personal control (Burger et al., 2011) and ontological security (Banham, 2020). As an “adaptive” response, the variety-seeking behavior may help consumers largely cope with sudden threats (Min and Schwarz, 2021). Future research should further explore this question.

Third, future research could explore variety-seeking behaviors in diversified consumption contexts. Current studies primarily examined purchasing or shopping for daily essentials (Choi et al., 2006; Shen and Wyer, 2010; Durante and Arsena, 2015; Gullo et al., 2019). Some scholars also tried to extend research scenarios to other consumption contexts, such as dining in restaurants (Huang and Kwong, 2016) and charitable donations (Levav and Zhu, 2009). Future researchers could investigate more variety-seeking behaviors in other common consumption behaviors in daily life, which lack attention. In addition, people could also have consumption behaviors in other situations, such as online shopping, purchasing service in massage shops, traveling across cities or countries, sporting goods purchases, or medical inquiries in the online community. The factors influencing consumers’ variety-seeking behaviors in such different situations have not been discussed in detail or sufficiently. This research gap provides an opportunity for scholars to introduce variety-seeking into the domains of e-marketing, service marketing, cause-related marketing, the online health community, and others. It is an essential step to enrich the current findings and provide novel research perspectives for other research fields.

Fourth, future research could explore variety-seeking behavior in the digital consumption world, which the current field has not fully discussed. As a digital platform to promote information sharing and user-created content, social media has innovated the way people connect, communicate, and develop relationships. The unique characteristics of social media may challenge the existing theories and frameworks explaining cognition, emotion, and behaviors (McFarland and Ployhart, 2015), meaning that future research on variety-seeking behavior should also consider the impact of the new media environment (Woolley and Sharif, 2022). For example, because people have anonymous perceptions, their communication on social media could avoid the negative influence of face-to-face connections. Future research can determine if social pressure from traditional communication still has the same effect on variety-seeking behaviors. Since social media provides more opportunities to share information across an extensive range of people, future studies can examine whether this broad mindset triggers variety-seeking behaviors. Furthermore, social media is an essential platform for companies to deliver brand information to target consumers, and future research could investigate the impact of brand display style in social media on variety-seeking behaviors in consumption.

Fifth, with the development and application of emerging technology in marketing (such as artificial intelligence, virtual reality, and augmented reality), future research could focus more on the relationship between these high-end technologies and variety-seeking behaviors. For instance, service robots may bring novelty experiences to consumers. Service robots in the consumption context may influence variety-seeking behaviors because the satisfaction of novelty and curiosity is a significant internal motivation for individuals seeking variety (McAlister, 1982). Service robots could bring novelty and curiosity or result in fear and rejection if anthropomorphic forms are overused (Mende et al., 2019). Consumers could adopt self-defense and protection mechanisms out of vigilance against fear and threats. Affected by a sense of identity threat, consumers may seek additional choices among similar commodities to avoid risks and make compensatory consumption (White et al., 2013). Meißner et al. (2020) explored how virtual reality affects consumer choice and found that consumers show more variety-seeking in high-immersive than low-immersive virtual reality. Future research could investigate the underlying mechanism of the effect of virtual reality on variety-seeking behaviors and how augmented reality could affect such behaviors (Rauschnabel et al., 2019).

Sixth, future research could consider solving inconsistencies in the existing literature, such as the effect of personal arousal level. Roehm and Roehm (2004) showed that people seek more variety at low arousal than high arousal moments. In contrast, Gullo et al. (2019) pointed out that individuals’ variety-seeking is lower in the early morning due to the lower arousal and stimulations. Another inconsistency is the effect of lack of personal control. Chang et al. (2021) found that failure weakens consumers’ perception of control, and consumers who have failed in a competition or not achieved a goal tend to seek less variety in subsequent consumption; however, according to compensatory consumption, prior research illustrated that variety-seeking as a compensatory strategy could restore the lack of personal control (Yoon and Kim, 2018; Huang et al., 2019). Thus, researchers could investigate the deeper mechanism and boundary conditions of these incongruent findings.

Last, future research requires more diversified research designs and data collections. Most studies measured variety-seeking behavior in consumption in the laboratory environment or adopted simulated or physical selections to explore consumers’ more real choice behavior. Furthermore, some scholars used actual shopping panel data to explore variety-seeking behavior in consumption at different times (Levav and Zhu, 2009; Yoon et al., 2011; Gullo et al., 2019); however, the current research on measuring variety-seeking behavior in consumption in the real environment is still insufficient. Researchers can increase their use of field experiments in future studies and explore more diverse and abundant physiological and behavioral data in real sales scenes to measure variety-seeking behavior in consumption. Additionally, more eye-tracking and neuromarketing EEG technologies also could be applied to obtain more accurate physiological data.

## Conclusion

Variety-seeking, as a common choice strategy for consumers, benefits market segmentation, promotion performance, and consumers’ welfare, which has led directly to the increase in academic research and studies in recent years (Koschate-Fischer et al., 2018; Gullo et al., 2019; Huang et al., 2019; Huang and Dong, 2019; Sela et al., 2019; Chang et al., 2021). The current article provides an intensive review of 61 identified papers in the marketing literature to understand how prior scholars explore the influencing factors of variety-seeking, investigate the underlying mechanism from distinct perspectives, and measure variety-seeking behaviors by various methods. These three parts are incorporated into a proposed research framework.

The influencing factors that researchers have adopted are classified into two categories: internal and external factors. Notably, internal factors have been widely discussed from five aspects: individual demographic, personality characteristics, emotion and physical state, sensory clues, and mindset. External factors involve three aspects at the present stage: social environment, physical environment, and marketing strategy, which are needed to extend. Thus, previous research is bound to various theoretical perspectives due to different influencing factors. Optimal stimulus level theory is a fundamental theory that has been widely applied in many studies to explain variety-seeking behavior. Other theoretical perspectives are also adopted to interpret variety-seeking behaviors in consumption, including personality traits, emotional coping, compensatory consumption, environmental psychology, and evolutionary psychology. These perspectives extend research fields of variety-seeking. Given measurement methods, survey scales are used to measure people’s intentions of variety-seeking, and scenario simulation is the most used approach to measure consumers’ variety-seeking in the experiment. Meanwhile, to observe variety-seeking behavior more objectively, researchers record participants’ real behaviors in experiments and analysis individuals’ real purchase behavior data from retailers.

Conversely, other important areas, such as digital consumption, emerging technology, and physiological measurement technology, have not received sufficient research attention, as well as other influencing factors and consumption contexts. Accordingly, this study identified several research gaps and proposed seven potential research directions for these areas. In addition, there are inconsistent findings in the existing literature. Future research could address these inconsistencies and provide explanations.

Overall, the contribution of this study is significant. Qualitatively, this paper conducted an intensive review of identified articles to reveal the influencing factors, theoretical perspectives, and measure methods of variety-seeking behavior in consumption and key findings, which can be used as an immediate reference for other researchers in this area. Quantitatively, this paper devised one research framework to incorporate the influencing factors, theoretical perspectives and underlying mechanisms, and measurement methods used in the 61 empirical studies, which provides a pictorial summary and enables readers to understand the body of research conducted on variety-seeking behavior in consumption. Further, this paper suggested seven future research directions, which may help researchers identify related topics in this subject area. The results of this study also have practical implications for the real world. Marketing managers could make segmentation based on internal factors, such as individual demographic and personality characteristics. Other internal factors, including emotion and physical state, sensory clues, and mindset, as well as external factors, could be manipulated in marketing activities, help to shape consumers’ variety-seeking behaviors and benefit promotion performance.

While this research has its merits, certain limitations remain. First, the review of the extant literature may not be exhaustive. More works are required to include relevant papers from different sources. Second, variety-seeking behavior in consumption is still in its concerning stage. Thus, additional journal papers with empirical results will continue to surface. More recently published variety-seeking research should be considered in future studies. Finally, in terms of article types, this paper focused on empirical studies, other conceptual or qualitative research is required.

## Author Contributions

The author confirms being the sole contributor of this work and has approved it for publication.

## Funding

Funding was provided by Huaqiao University’s Academic Project Supported by the Fundamental Research Funds for the Central Universities (21SKGC-QG05).

## Conflict of Interest

The author declares that the research was conducted in the absence of any commercial or financial relationships that could be construed as a potential conflict of interest.

## Publisher’s Note

All claims expressed in this article are solely those of the authors and do not necessarily represent those of their affiliated organizations, or those of the publisher, the editors and the reviewers. Any product that may be evaluated in this article, or claim that may be made by its manufacturer, is not guaranteed or endorsed by the publisher.
